# Brain barriers and their potential role in migraine pathophysiology

**DOI:** 10.1186/s10194-021-01365-w

**Published:** 2022-01-26

**Authors:** Astrid Wiggers, Håkan Ashina, Nouchine Hadjikhani, Abhay Sagare, Berislav V. Zlokovic, Martin Lauritzen, Messoud Ashina

**Affiliations:** 1 Danish Headache Center, Department of Neurology, Rigshospitalet Glostrup, Faculty of Health and Medical Sciences, University of Copenhagen, Valdemar Hansens Vej 5, DK-2600 Glostrup, Denmark; 2 Department of Neurorehabilitation and Traumatic Brain Injury, Rigshospitalet, Kettegaards Allé 30, 2650 Hvidovre, Copenhagen, Denmark; 3 Martino Center for Biomedical Imaging, Massachusetts General Hospital, Harvard Medical School, 149 Thirteenth Street, Charlestown, MA USA; 4 Department of Physiology and Neuroscience and the Zilkha Neurogenetic Institute, Keck School of Medicine of the University of Southern California, 1501 San Pablo Street, California, Los Angeles 90089 USA; 5 Department of Neuroscience, Faculty of Health Sciences, University of Copenhagen, Blegdamsvej 3B, DK-2200 Copenhagen N, Denmark

**Keywords:** Headache, Trigeminovascular system, Blood-brain barrier, Aura

## Abstract

Migraine is a ubiquitous neurologic disease that afflicts people of all ages. Its molecular pathogenesis involves peptides that promote intracranial vasodilation and modulate nociceptive transmission upon release from sensory afferents of cells in the trigeminal ganglion and parasympathetic efferents of cells in the sphenopalatine ganglion. Experimental data have confirmed that intravenous infusion of these vasoactive peptides induce migraine attacks in people with migraine, but it remains a point of scientific contention whether their site of action lies outside or within the central nervous system. In this context, it has been hypothesized that transient dysfunction of brain barriers before or during migraine attacks might facilitate the passage of migraine-inducing peptides into the central nervous system. Here, we review evidence suggestive of brain barrier dysfunction in migraine pathogenesis and conclude with lessons learned in order to provide directions for future research efforts.

## Introduction

Migraine is a prevalent neurological disorder that is characterized by recurrent headache attacks of moderate to severe intensity and accompanying symptoms such as nausea, vomiting, photo-, and phonophobia [1]. Its pathogenesis is to be explained within the framework of the trigeminovascular system [2]. This system includes the trigeminal ganglion and its peripheral axonal projections that innervate pain-sensitive intracranial structures, e.g. meninges [3]. In addition, central axonal projections arise from trigeminal ganglion cells and convey nociceptive impulses to second-order trigeminovascular neurons in the brain stem [3]. These neurons, in turn, project to third order trigeminovascular neurons in the thalamus, which then convey nociceptive impulses to a wide array of cortical areas that are involved in pain processing, e.g. the somatosensory cortex [3].

A point of scientific contention is whether the molecular mechanisms that initiate migraine attacks lie outside or within the central nervous system (CNS) [1]. Upon activation, peripheral projections of the trigeminal nerve release neurotransmitters that elicit vasodilation and modulate nociceptive transmission, e.g. calcitonin gene-related peptide (CGRP) and pituitary adenylate cyclase-activating polypeptide (PACAP) [3]. Intravenous administration of these neurotransmitters can induce migraine attacks in individuals with migraine, whereas healthy volunteers most often develop no more than mild headache [4]. Based on this, it becomes a question of key interest whether these neuropeptides can cross the blood-brain barrier (BBB) and initiate migraine attacks from within the CNS. If not, this would favor a peripheral origin of migraine.

In this Review, we examine evidence suggestive of brain barrier dysfunction in migraine. Furthermore, we discuss whether neuropeptides that induce migraine attacks have their site of action within the CNS. Lastly, we review some of the outstanding research questions and provide directions for future research efforts.

### Brain barriers

The brain has multiple barriers to restrict non-selective passage of solutes into brain parenchyma [5]. In the meninges, the arachnoid barrier impedes the leakage of solutes from fenestrated blood vessels into the subarachnoid space that is filled with cerebrospinal fluid (CSF). Blood vessels in the subarachnoid space consist of endothelial cells that are connected by tight junctions with similar barrier characteristics as blood vessels in brain parenchyma but without surrounding pericytes and astrocytic end-feet [6]. This hinders passage of solutes from the blood to the CSF and is called the blood-CSF-barrier (BCSFB). The arterioles that branch from the subarachnoid blood vessels penetrate the brain parenchyma and constitute the brain microvasculature. The microvasculature is part of the BBB, a dynamic interface comprised of vascular cells (e.g. endothelium, pericytes), glial cells (e.g. astrocytes), and neurons [5–7].

As a rule of thumb, hydrophilic molecules of less than 620 Da cross the BBB via diffusion along the paracellular route, and small lipophilic molecules diffuse freely through the lipid membranes. However, the majority of these freely diffusing lipophilic molecules are rapidly removed from endothelial cells by efflux transporters and do not reach the brain parenchyma. All other solutes require transporters located on endothelial cells [5]. Solutes also enter the CNS via the circumventricular organs (CVOs) that are free of BBB and located near the ventricular system [8]. The CVOs include the following structures: area postrema, median eminence, pineal gland, pituitary gland, subcommissural organ, subfornical organ, and vascular organ of lamina terminalis [8]. Their leakiness allows accumulation of circulating agents, but a barrier comprised of tanycytes with tight junctions prevents the passage of agents into the CSF [6].

### Blood-brain barrier dysfunction in migraine pathogenesis

The hypothesis of BBB dysfunction in migraine was first proposed by Harper and colleagues in 1977 [9]. The authors speculated that a leaky BBB allowed circulating agents in the peripheral blood to enter the CNS and facilitate transmission of nociceptive impulses that ultimately yield the perception of migraine pain. However, there is currently very limited experimental evidence in favor of this hypothesis. Three magnetic resonance imaging (MRI) studies found no evidence of a leaky BBB during and outside of spontaneous migraine attacks (Table 1) [10–12]. Two of the studies used gadolinium-based dynamic contrast-enhanced (DCE) MRI to assess disruption of the BBB in five regions of interest, being the anterior, middle, and posterior cerebral area, brain stem, posterior pons, and whole brain [10, 11]. Patients were scanned during a spontaneous migraine attack as well as on an attack-free day. No changes suggestive of BBB dysfunction were identified in 19 patients with migraine without aura [14] or 19 patients with migraine with aura [15] when comparing data during and outside of migraine attacks. There was also no association between BBB permeability and any headache feature (e.g., location, intensity). However, post hoc power analysis showed that BBB permeability changes of less than 35% in patients with migraine without aura and changes of less than 11% in patients with migraine with aura could not be excluded [10, 11]. Another limitation is that early and/or transient changes in BBB permeability may not have been detected, as median time from onset of attack to MRI scan was 6.5 h in patients with migraine without aura [14] and 7.6 h in patients with migraine with aura [15]. In a third DCE-MRI study, differences in BBB permeability were assessed in 35 patients with migraine with/without aura and 21 healthy non-headache controls [12]. Patients with migraine were scanned outside of attacks and the authors found no changes in BBB permeability when comparing the two groups. Although they did find a lower fractional plasma volume in the left amygdala of patients with migraine when compared with healthy controls [12], it is unclear whether this finding has any relevance to BBB dysfunction during migraine attacks.

**Table 1:** Human experimental studies of BBB integrity in migraine

Study	Method	Study population	Outcomes	Limitations
Amin et al., 2017 [10]	Gadolinium-based-DCE-MRI at rest and during spontaneous migraine attacks. Permeability assessed in five different brain regions located in the anterior, middle, and posterior cerebral area, brain stem and posterior pons.	19 MO	No changes in BBB permeability on attack versus headache-free days. No changes in BBB permeability between pain and non-pain side.	Power of study caused a detection limit of 35% Permeability assessed using a 604 Da extremely hydrophilic molecule Median time of onset of attack to scan was 6.5 h.
Hougaard et al. 2017 [11]	Gadolinium-based-DCE-MRI at rest and during spontaneous migraine attacks. Permeability assessed in five different brain regions located in the anterior, middle, and posterior cerebral area, brain stem and posterior pons.	19 MA	No changes in BBB permeability on attack versus headache-free days. No changes in BBB permeability between pain and non-pain side. No difference in affected or non-affected hemispheres.	Power of study caused a detection limit of 11% Permeability assessed using a 604 Da extremely hydrophilic molecule. Median time of onset of attack to scan was 7.6 h and no patients were scanned during aura symptoms.
Kim et al., 2019 [12]	Gadolinium-based-DCE-MRI was performed on migraine patients outside of attacks and compared with scans of healthy controls	21 MA 14 MO 21 Healthy controls	No difference in gadolinium BBB permeability between patients and controls. Lower fractional plasma volume in left amygdala in migraine patients	Permeability assessed using a 604 Da extremely hydrophilic molecule Age of control group was not matched with migraine group Changes in amygdala cannot be directly correlated to changes in BBB integrity
Schankin et al., 2016 [13]	PET-scan and the radioligand ^11^C-dihydroergotamine at rest and during GTN-induced migraine attacks.	2 MA 4 MO 6 Heathy controls	No binding of the radioligand to brain parenchyma at rest or during GTN-induced attacks in migraineurs or healthy controls.	Limited spatial resolution of PET Permeability assessed with ^11^C-DHE with a molecular size of 583.7 g/mol. GTN-induced headache instead of spontaneous

*BBB* Blood-Brain Barrier, *Da* Dalton, *DCE-MRI* Dynamic Contrast-Enhanced Magnetic Resonance Imaging, *GTN* Glyceryl trinitrate, *H* Hour, *MO* Migraine without aura, *MA* Migraine with aura, *PET* Positron Emission Tomography

BBB permeability has also been assessed during provoked migraine attacks using positron emission tomography – computed tomography (PET-CT) with the radioligand ^11^C-dihydroergotamine (^11^C-DHE) (Table 1) [13]. Migraine attacks were induced by intravenous infusion of the nitric oxide donor glyceryl trinitrate (GTN) which is a potent vasodilator known to provoke migraine attacks in 80% of patients with migraine [16]. It should be noted that patients were eligible for study inclusion only if they developed a migraine attack after GTN infusion whereas subjects in the control group had to remain free of pain following GTN infusion [13]. The authors reported no changes suggestive of BBB dysfunction when comparing scans before and during provoked attacks, or when comparing scans of patients to those of controls. However, the limited spatial resolution of PET and the usage of ^11^C-DHE tracer (584 Da) might impede the detection of minor changes in BBB permeability [13]. Taken together, it seems evident that neuroimaging studies provide no evidence for BBB dysfunction during migraine attacks, although early transient or minor changes in BBB permeability cannot be fully excluded.

Dysfunction of the BBB has been evaluated by the activity of matrix metallopeptidases (MMPs) since some members of this protease family seem to be implicated in breakdown of the BBB [17]. In a rodent study, cortical spreading depression (CSD) led to BBB disruption and an increase in MMP-9 levels in cortical homogenates ipsilateral to the induced CSD [18]. However, CSD was induced by three pinpricks after removing large parts of the calvarium bilaterally and opening the dura mater. This procedure had evidently caused neuroinflammatory responses which, in turn, limits the significance of the study findings. Similarly, there is conflicting data from studies that have assessed plasma MMP-9 levels in human subjects. Some studies report elevated plasma MMP-9 levels in migraine patients compared with controls [14, 15] while others find no association between plasma MMP-9 levels and migraine [19, 20]. Thus, it is not possible to draw any firm conclusions based on measurements of plasma MMP-9. A few limitations should also be noted. First, MMPs are produced by various cell types inside and outside the nervous system. It is therefore unknown whether MMPs that are produced in intracerebral cells reach the peripheral circulation. Second, MMP measurements are not a specific measure of BBB dysfunction since intracerebral levels of MMP-3 and MMP-9 expression were elevated in an animal model of epileptic seizures while no changes in BBB permeability were observed [21]. Lastly, elevated levels of MMP-9 have been reported in various disorders that are not presumed to have alterations in BBB permeability, e.g. idiopathic atrial fibrillation [22] and rheumatoid arthritis [23].

An aspect that merits emphasis is the special case of migraine with aura. CSD is widely recognized as the neurobiological substrate of aura and is characterized by a self-propagating cortical wave of electrophysiological hyperactivity following by inhibition [24].

Based on animal data, it seems evident that CSD induces inflammatory processes within the brain and meninges which, in turn, appears to increase the firing rate of first and second order trigeminovascular neurons [25–27]. A recent PET-MRI study using the ligand ^11^C-PBR28 observed strong extra-axial inflammatory signals in the meninges overlying the occipital lobe during migraine with visual aura in 11 migraine patients [28]. Repetitive episodes of neuroinflammation in migraine patients could result in a leaky BBB and allow passage of neuropeptides into the brain parenchyma [29, 30]. Further studies are needed to evaluate whether CSD-induced inflammatory processes are associated with changes in brain barrier permeability.

### Provoked migraine attacks

The trigeminovascular system is widely considered the anatomical and physiological substrate of migraine pathogenesis [1]. Within this framework, parasympathetic efferents of cells in the sphenopalatine ganglion and sensory afferents of cells in the trigeminal ganglion release, upon activation, various peptides that promote dilation of intracranial arteries and modulate nociceptive transmission [1]. Decades of research have established that intravenous infusion of certain naturally occurring peptides can induce migraine attacks in patients with migraine while healthy volunteers develop most often no more than a mild headache [4]. This raises the question of whether these peptides induce migraine attacks outside or within the CNS.

The following peptides have been implicated in migraine pathogenesis [31]: adrenomedullin (ADM), amylin, calcitonin gene-related peptide (CGRP), pituitary adenylate cyclase-activating polypeptide (PACAP), and vasoactive intestinal polypeptide (VIP). All are potent vasodilators and induce migraine attacks when administered by intravenous infusion to patients with migraine [31, 32]. They mediate their effects via G protein-coupled receptors that, in turn, activate the cyclic adenosine monophosphate (cAMP)-dependent signaling pathway [31]. Preclinical evidence suggests that this pathway results in the opening of ATP-sensitive potassium (K_ATP_) channels, and it has been hypothesized that opening of potassium channels might be the final common pathway in the genesis of a migraine pain [1]. Collectively, the neuropeptides have receptor-binding sites that are expressed at multiple levels of the trigeminovascular system (Table 2) of which the extracerebral vasculature, extracranial vasculature and the trigeminal ganglion is not brain barrier protected.

**Table 2:**
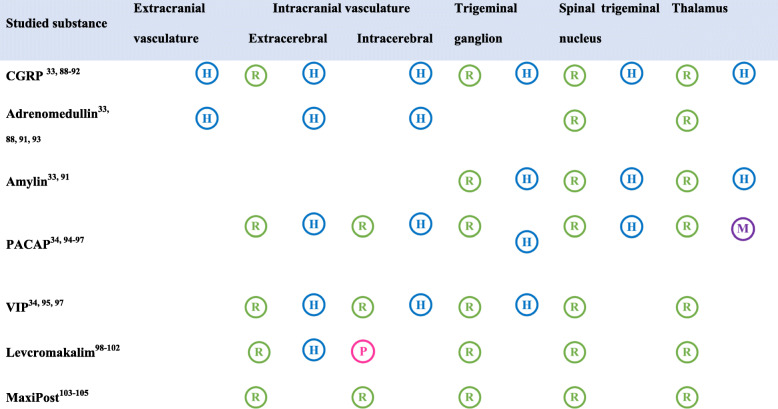
Receptor binding sites within the trigeminovascular system. The table gives an overview of seven different migraine-inducing substances and their various binding sites within the trigeminovascular system. In this table, the trigeminovascular system is divided into the following structures: extracranial vasculature, intracranial vasculature, the trigeminal ganglion, the spinal trigeminal nucleus, and thalamus. The binding sites have been detected by usage of polymerase chain reaction, in-situ hybridization, western blot, or immunostaining in human , monkey , pig , or rodent tissues

*CGRP* Calcitonin Gene-Related Peptide, *PACAP* Pituitary adenylate cyclase-activating peptide, *VIP* Vasoactive Intestinal Peptide

Direct binding of neuropeptides to Aδ-fibers or neurons in the trigeminal ganglion and subsequent hyperexcitability has been suggested as the pain-initiating mechanism in migraine. However, based on the suggested intracellular pathway with K_ATP_ channels as the end station direct binding to nerve fibers would result in hyperpolarization, and thus the vasculature might be a more relevant site of action. Other ganglia without barrier protection may also be involved in migraine pathogenesis, and preclinical data has suggested that activation of the sphenopalatine ganglion causes release of PACAP and VIP from its efferent fibers [1]. This mechanism is bypassed in provoked migraine attacks where the neuropeptides are given intravenously but could play a role in spontaneous attacks. It merits emphasis that ADM, amylin, and CGRP belong to the same family of peptides [33]. The same is also true for PACAP and VIP [34].

The molecular size of the aforementioned peptides suggests a very limited ability to cross the brain barrier (Table 3) [5]. This accords with animal studies that have radiolabeled all peptides, except for CGRP, and quantified the degree of BBB passage [40, 41, 43, 50, 69–71]. In rodents, injection of radiolabeled ADM does not cross the BBB [69], whilst the peak brain uptake of injected radiolabeled amylin is 0.11–0.13% [40, 70]. Furthermore, injection of radiolabeled PACAP yields a brain uptake of less than 0.07% in rodents for both its isoforms (i.e. PACAP-38 and PACAP-27) [43], while injection of radiolabeled VIP results in brain uptake of 0.15% in mice and no brain uptake in rats [41, 50].

**Table 3:** Brain Barrier Permeability of Migraine-Inducing Substances

Substance	Size (Dalton)	Permeability	Migraine induction rate	MCA changes in rodents	MCA changes in humans		
				In vitro Changes after luminal administration in vascular models	In vitro Changes after luminal administration in vascular models	In vivo Assessed by ultrasound and SPECT	In vivo Assessed by MRA
CGRP	3791.3	Unknown	57% [31]	No dilation [35]	Unknown	MCA velocity drop [36] rCBF no changes [36]	No changes [37]
Adrenomedullin	6028.8	Unknown	55% [38]	No dilation [35]	Unknown	MCA no changes [39] rCBF no changes [39]	Unknown
Amylin	3904.5	0.11–0.13% Inj/g brain (rodents) [40, 41]	41% [42]	Weak dilatory response [35]	Unknown	Unknown	Unknown
PACAP27	3147.6	0.066% in brain parenchyma (rodents) [43]	55% [44]	No dilation [45]	Unknown	Unknown	No changes [46]
PACAP38	4534.3	0.053% in brain parenchyma (rodents) [43]	58% [47]	No dilation [45, 48]	No dilation [35]	MCA velocity drop [47] rCBF not measured [47]	No changes [49]
VIP	3326.8	None (rodents) [50] 0.15% Inj/g brain (rodents) [41]	71% [32]	No dilation [45, 48]	No dilation [35]	MCA velocity drop [51, 52] rCBF no changes [51]	No changes [53]
GTN	227.09	Yes	80% [16]	Unknown	Unknown	MCA velocity drop [54] rCBF no changes [54]	Dilation [55, 56]
Sildenafil	474.6	0.028% Inj/g brain (rodents) [57]	83% [58]	No dilation [59, 60]	Unknown	MCA no changes [58] rCBF no changes [58]	Unknown
Cilostazol	369.5	Yes	86% [61], 88% [62]	Unknown	Unknown	MCA velocity drop [63] rCBF no changes [63]	Dilation [64]
Levcromakalim	286.3	Yes	100% [65]	Unknown	Unknown	Unknown	Dilation [66]
MaxiPost	359.7	Yes	95% [67]	Unknown	Unknown	MCA velocity drop [68] rCBF not measured [68]	Unknown

*CGRP* Calcitonin Gene-Related Peptide, *GTN* Glyceryl Trinitrate, *Inj/g* Injection/g, *MCA* Middle Cerebral Artery, *MRA* Magnetic Resonance Angiography, *PACAP27* Pituitary adenylate cyclase-activating peptide 27, *PACAP38* Pituitary adenylate cyclase-activating peptide 38, *rCBF* Regional Cerebral Blood Flow, *SPECT* Single Photon Emisson Computed Tomography, *VIP* Vasoactive Intestinal Peptide. Molecular sizes obtained from PubChem (pubchem.ncbi.nlm.nih.gov)

Another method to investigate possible brain barrier passage of migraine-inducing peptides involves the study of their vascular responses. In this context, dilation of the middle cerebral artery (MCA) has often been used as a surrogate marker of BBB penetration. The MCA is surrounded by the BCSFB and branches into smaller vessels that penetrate the brain parenchyma. These small vessels are surrounded by the BBB and constitute the cerebral microvasculature. Based on this, it is assumed that lack of MCA dilation by migraine-inducing peptides would suggest no or very limited BBB passage. This, in turn, would indicate that the site of action of migraine-inducing peptides is located outside of the CNS.

The vascular responses of migraine-inducing peptides have been extensively studied in vitro using rodent or human MCA. These studies have found that ADM, CGRP, both PACAP isoform, and VIP did not elicit dilation of the rat MCA following luminal application [35, 45, 48, 59], whilst amylin induced only a weak dilator response [71]. Furthermore, luminal application of both PACAP isoforms and VIP did not evoke dilation of the human MCA [45, 71]. Although these preclinical studies provide evidence against a central side of action of migraine-inducing substances, it should be recognized that tissue preparation can affect transporters located in the endothelium which, in turn, might affect tissue permeability.

This issue is avoided when assesing vasodilation in vivo by using ultrasound or magnetic resonance angiography (MRA). Ultrasound of brain arteries detects changes in blood velocity, a factor inversely proportional to the diameter of the blood vessel. Since the blood flow is also dependent on the vascular diameter, a decrease in MCA velocity only reflects vasodilation if the single photon-emission computed tomography (SPECT)-determined regional cerebral blood flow (rCBF) is unchanged [54].

A decrease in ultrasound assessed MCA velocity and no change in SPECT-determined rCBF has been reported after CGRP and VIP infusion [36, 51] whereas infusion of ADM did not affect any of the two parametres [39]. This suggests CGRP- and VIP-induced vasodilation of the MCA and possibly BCSFB passage. A decrease in MCA velocity was also reported after infusion of PACAP-38, and PACAP-38 has previously been reported not to affect rCBF in healthy volunteers [47, 72]. Although some of the ultrasound-based studies indicate BCSFB permeability, the results should be interpreted with caution since this method assesses vasodilation indirectly.

MRA enables visualization of extra- and intracerebral arteries and direct measurement of arterial circumferences. MRA studies performed on healthy controls and migraine patients report dilation of the MMA after infusion of CGRP, PACAP-27, PACAP-38, and VIP but no change of the MCA circumference [37, 46, 49, 53, 73]. This is consistent with no BCSFB passage and suggestive of a peripheral site of action. Lack of brain barrier permeability of the neuropeptides might also explain the lack of CNS side effects in human experimental studies with intravenous infusion of migraine-inducing peptides. It should also be noted that CGRP infusion did not modulate blood-oxygen-level-dependent (BOLD) responses in the visual cortex of healthy volunteers which is consistent with no or very limited passage of CGRP across the BBB [74]. Taken together, the available experimental data favors the assertion that migraine-inducing neuropeptides bind to their receptors outside of the BBB.

### Therapies targeting CGRP-signaling

The recent advent of small molecule CGRP receptor antagonists, gepants, and monoclonal antibodies (mAbs) targeting CGRP or its receptor have expanded the therapeutic armamentarium for migraine. Important questions have since been raised on whether these drugs can cross the BBB and exert their therapeutic effects from within the CNS [75]. This seems unlikely based on the available data from an in vivo PET study in which the authors reported very low human CGRP receptor occupancy following administration of telcagepant at an efficacious dose – suggestive of a peripheral site of action for telcagepant [76]. This finding accords well with the observation of reduced mechanical sensitivity thresholds in rodents following intraperitoneal, but not intracerebroventricular, injection of olcegepant and a mAb against CGRP [77]. Furthermore, intravenous injection of fluorescently-labeled fremanezumab yielded labeling of sensory and autonomic ganglia as well as the dura mater, whereas no fluorescent signal was observed in structures within the CNS [78].

Collectively, it seems evident that therapies targeting CGRP signaling are unlikely to cross the BBB which, in turn, indicates that BBB passage is not needed to achieve therapeutic benefits with medications for migraine. It might indeed be advisable to develop drugs that do not cross the BBB to avoid adverse effects associated with CNS depression. For example, lasmiditan (serotonin (5-HT) 1F receptor agonist) is an acute medication for migraine that can cause CNS-related side effects (incl. Dizziness, sedation, and temporary driving impairment) which are likely to limit its use in clinical practice [79–81].

### Outstanding research questions

The current evidence obtained from both neuroimaging and biochemical markers in humans suggests no disruption of the brain barriers in migraine. However, the limited sensitivity of the applied methods requires more studies to assess the relationship between brain barrier dysfunction and migraine pathophysiology. Future studies could use the newly developed sensitive modified DCE-MRI method that considers the arterial input function and cerebral blood flow [82] since both these parameters could be affected in migraine. This method has identified BBB dysfunction in early stages of cognitive dysfunction [82]. Additionally, soluble PDGFRβ, a biomarker of BBB pericyte injury, could be analyzed in migraine patients [83].

The limited brain barrier passage of migraine-inducing neuropeptides suggests a peripheral origin of migraine. However, migraine attacks can also be induced in migraine patients by administration of vasoactive molecules with BBB permeability (e.g. GTN or cilostazol [16, 61, 62]), and several questions concerning migraine origin remain unanswered. One of them is the presence of premonitory symptoms (PS) in migraine which might be suggestive of initial activation of central structures in migraine attacks. The underlying mechanisms of PS are still unclear. Infusion of GTN to migraine patients induced PS in 36% (12/33) of patients prior to triggered migraine attacks [84]. In another study, GTN was found to induce PS in a selected group of patients known to have migraine with PS while PET-scans showed activation in various different brain areas, including hypothalamus [85]. In this study, however, no control group was included, and thus changes may relate to GTN administration rather than migraine. Furthermore, none of these studies compared PS in patients who reported and did not report migraine attacks. A study assessing the incidence of PS in migraine patients after administration of trigeminal signaling molecules reported no PS after CGRP infusion but PS in 48% of patients after PACAP-38 infusion [86]. However, CGRP and PACAP38 did not induce more PS in patients who developed an attack compared to those who did not develop an attack [86], and this aspect must be studied in healthy subjects. Further studies are needed to clarify the presence of a premonitory phase in migraine which may contribute to the discussion of migraine origin.

Additionally, several outstanding questions relate to migraine aura. Although CSD is accepted as the substrate of migraine aura, it is still unknown how CSD arises in a seemingly otherwise healthy cerebral cortex of migraine patients, and how it is related to the headache phase of migraine. The unpredictable and short-lasting nature of migraine aura makes it difficult to study patients during symptoms and thereby answer outstanding research questions on this matter. However, recently a randomized, double-blind, placebo-controlled, crossover study reported that administration of the K_ATP_-channel opener levcromakalim induced aura in 10 of 17 (59%) patients suffering from migraine with aura and migraine attacks in 14 of 17 (82%) the patients [87]. The authors suggest that K_ATP_-channel opening most likely induces CSD and migraine headache via separate pathways since levcromakalim efficiently triggers migraine without aura [65] and this even in some patients who have previously experienced aura symptoms during all their migraine attacks [87]. However, the trigger of migraine aura is still unknown and future research efforts are required to fully understand the initiation CSD and its relation to the headache phase of migraine.

## Conclusion

Brain barrier disruption has been hypothesized to play an important role in the genesis of migraine attacks. The current evidence suggests, however, that there is limited experimental data in favor of this hypothesis. Nonetheless, it cannot be excluded that, in particular, CSD might be associated with inflammatory processes within the brain and meninges, ultimately causing transient brain barrier disruption. Further studies are warranted to ascertain whether early transient changes in BBB permeability occur during the early phases of a migraine attack.

## Data Availability

Not applicable.

## Abbreviations

CNS: Central nervous system
CGRP: Calcitonin gene-related peptide
PACAP: Pituitary adenylate cyclase-activating peptide
BBB: Blood-brain barrier
CSF: Cerebrospinal fluid
BCSFB: Blood-cerebrospinal fluid barrier
CVO: Circumventricular organs
MRI: Magnetic resonance imaging
DCE MRI: Dynamic contrast-enhanced magnetic resonance imaging
PET-CT: Position emission tomography-computed tomography
GTN: Glyceryl trinitrate
^11^C-DHE: ^11^C-Dihydroergotamine
MMP: Matrix metallopeptidase
CSD: Cortical spreading depression
ADM: Adrenomedullin
VIP: Vasoactive intestinal peptide
cAMP: Cyclic adenosine monophosphate
K_ATP_: ATP-sensitive potassium channel
MCA: Middle cerebral artery
MRA: Magnetic angiography
SPECT: Single-photon emission computerized tomography
rCBF: Regional cerebral blood flow
BOLD: Blood-oxygen-level-dependent
PS: Premonitory symptoms
mAb: monoclonal antibody

## References

[1] Ashina M, Ropper AH (2020). Migraine. N Engl J Med.

[2] Moskowitz MA (1984). The neurobiology of vascular head pain. Ann Neurol.

[3] Ashina M, Hansen JM, Do TP, Melo-Carrillo A, Burstein R, Moskowitz MA (2019). Migraine and the trigeminovascular system—40 years and counting. Lancet Neurol.

[4] Ashina M, Hansen JM, BO AD, Olesen J. (2017). Human models of migraine - short-term pain for long-term gain. Nat Rev Neurol.

[5] Banks WA (2016). From blood-brain barrier to blood-brain interface: new opportunities for CNS drug delivery. Nat Rev Drug Discov.

[6] Saunders NR, Dziegielewska KM, Møllgård K, Habgood MD (2018). Physiology and molecular biology of barrier mechanisms in the fetal and neonatal brain. J Physiol.

[7] Kutuzov N, Flyvbjerg H, Lauritzen M (2018). Contributions of the glycocalyx, endothelium, and extravascular compartment to the blood–brain barrier. Proc Natl Acad Sci U S A.

[8] Ermisch A, Brust P, Kretzschmar R, Rühle HJ (1993). Peptides and blood-brain barrier transport. Physiol Rev.

[9] Harper AM, MacKenzie ET, McCulloch J, Pickard JD (1977). Migraine and the blood-brain barrier. Lancet..

[10] Amin FM, Hougaard A, Cramer SP, Christensen CE, Wolfram F, Larsson HBW, Ashina M (2017). Intact blood-brain barrier during spontaneous attacks of migraine without aura: a 3T DCE-MRI study. Eur J Neurol.

[11] Hougaard A, Amin FM, Christensen CE, Younis S, Wolfram F, Cramer SP, Larsson HBW, Ashina M (2017). Increased brainstem perfusion, but no blood-brain barrier disruption, during attacks of migraine with aura. Brain..

[12] Kim YS, Kim M, Choi SH, You SH, Yoo RE, Kang KM, Yun TJ, Lee ST, Moon J, Shin YW (2019). Altered vascular permeability in migraine-associated brain regions: evaluation with dynamic contrastenhanced MRI. Radiology..

[13] Schankin CJ, Maniyar FH, Seo Y, Kori S, Eller M, Chou DE, Blecha J, Murphy ST, Hawkins RA, Sprenger T, VanBrocklin HF, Goadsby PJ (2016). Ictal lack of binding to brain parenchyma suggests integrity of the blood-brain barrier for 11C-dihydroergotamine during glyceryl trinitrate-induced migraine. Brain..

[14] Leira R, Sobrino T, Rodríguez-Yáñez M, Blanco M, Arias S, Castillo J (2007). MMP-9 immunoreactivity in acute migraine. Headache..

[15] Imamura K, Takeshima T, Fusayasu E, Nakashima K (2008). Increased plasma matrix metalloproteinase-9 levels in migraineurs. Headache..

[16] Thomsen LL, Kruuse C, Iversen HK, Olesen J (1994). A nitric oxide donor (nitroglycerin) triggers genuine migraine attacks. Eur J Neurol.

[17] Rosenberg GA, Cunningham LA, Wallace J, Alexander S, Estrada EY, Grossetete M, Razhagi A, Miller K, Gearing A (2001). Immunohistochemistry of matrix metalloproteinases in reperfusion injury to rat brain: activation of MMP-9 linked to stromelysin-1 and microglia in cell cultures. Brain Res.

[18] Gursoy-Ozdemir Y, Qiu J, Matsuoka N, Bolay H, Bermpohl D, Jin H, Wang X, Rosenberg GA, Lo EH, Moskowitz MA (2004). Cortical spreading depression activates and upregulates MMP-9. J Clin Invest.

[19] Martins-Oliveira A, Speciali JG, Dach F, Marcaccini AM, Gonçalves FM, Gerlach RF, Tanus-Santos JE (2009). Different circulating metalloproteinases profiles in women with migraine with and without aura. Clin Chim Acta.

[20] Ashina M, Tvedskov JF, Lipka K, Bilello J, Penkowa M, Olesen J (2010). Matrix metalloproteinases during and outside of migraine attacks without aura. Cephalalgia..

[21] Penkowa M, Florit S, Giralt M, Quintana A, Molinero A, Carrasco J, Hidalgo J (2005). Metallothionein reduces central nervous system inflammation, neurodegeneration, and cell death following kainic acid-induced epileptic seizures. J Neurosci Res.

[22] Li M, Yang G, Xie B, Babu K, Huang C (2014). Changes in matrix metalloproteinase-9 levels during progression of atrial fibrillation. J Int Med Res.

[23] Gruber BL, Sorbi D, French DL, Marchese MJ, Nuovo GJ, Kew RR, Arbeit LA (1996). Markedly elevated serum MMP-9 (gelatinase B) levels in rheumatoid arthritis: a potentially useful laboratory marker. Clin Immunol Immunopathol.

[24] Lauritzen M (1994). Pathophysiology of the migraine aura: the spreading depression theory. Brain..

[25] Karatas H, Erdener SE, Gursoy-Ozdemir Y (2013). Spreading depression triggers headache by activating neuronal Panx1 channels. Science.

[26] Zhang XC, Levy D, Noseda R, Kainz V, Jakubowski M, Burstein R (2010). Activation of meningeal nociceptors by cortical spreading depression: implications for migraine with aura. J Neurosci.

[27] Zhang XC, Levy D, Kainz V, Noseda R, Jakubowski M, Burstein R (2011). Activation of central TGV neurons by CSD. Ann Neurol.

[28] Hadjikhani N, Albrecht DS, Mainero C, Ichijo E, Ward N, Granziera C, Zürcher NR, Akeju O, Bonnier G, Price J, Hooker JM, Napadow V, Nahrendorf M, Loggia ML, Moskowitz MA (2020). Extra-axial inflammatory signal in Parameninges in migraine with visual Aura. Ann Neurol.

[29] Liktor-Busa E, Blawn KT, Kellohen KL (2020). Functional NHE1 expression is critical to blood brain barrier integrity and sumatriptan blood to brain uptake. PLoS One.

[30] Ghaffari H, Grant SC, Petzold LR, Harrington MG (2020). Regulation of CSF and brain tissue sodium levels by the blood-CSF and blood-brain barriers during migraine. Front Comput Neurosci.

[31] Ashina M, Terwindt GM, Al-Karagholi MA-M (2021). Migraine: disease characterisation, biomarkers, and precision medicine. Lancet.

[32] Pellesi L, Al-Karagholi MA-M (2021). Effect of Vasoactive Intestinal Polypeptide on Development of Migraine Headaches A Randomized Clinical Trial. JAMA Netw Open.

[33] Hendrikse ER, Bower RL, Hay DL, Walker CS (2019). Molecular studies of CGRP and the CGRP family of peptides in the central nervous system. Cephalalgia..

[34] Sundrum T, Walker CS (2017). Pituitary adenylate cyclase-activating polypeptide receptors in the trigeminovascular system: Implications for migraine. Br J Pharmacol.

[35] Edvinsson L, Nilsson E, Jansen-Olesen I (2007). Inhibitory effect of BIBN4096BS, CGRP 8-37, a CGRP antibody and an RNA-Spiegelmer on CGRP induced vasodilatation in the perfused and non-perfused rat middle cerebral artery. Br J Pharmacol.

[36] Lassen LH, Jacobsen VB, Haderslev PA, Sperling B, Iversen HK, Olesen J, Tfelt-Hansen P (2008). Involvement of calcitonin gene-related peptide in migraine: regional cerebral blood flow and blood flow velocity in migraine patients. J Headache Pain..

[37] Asghar MS, Hansen AE, Kapijimpanga T, van der Geest RJ, van der Koning P, Larsson HBW, Olesen J, Ashina M (2010). Dilation by CGRP of middle meningeal artery and reversal by sumatriptan in normal volunteers. Neurology..

[38] Ghanizada H, Al-Mahdi Al-Karagholi M, Arngrim N (2021). Effect of Adrenomedullin on migraine-like attacks in patients with migraine. Neurology..

[39] Petersen KA, Birk S, Kitamura K, Olesen J (2009). Effect of adrenomedullin on the cerebral circulation: relevance to primary headache disorders. Cephalalgia..

[40] Banks WA, Kastin AJ, Maness LM, Huang W, Jaspan JB (1995). Permeability of the blood-brain barrier to amylin. Life Sci.

[41] Dogrukol-Ak D, Banks WA, Tuncel N, Tuncel M (2003). Passage of vasoactive intestinal peptide across the blood-brain barrier. Peptides..

[42] Ghanizada H, Al-Karagholi MAM, Walker CS (2021). Amylin analog Pramlintide induces migraine-like attacks in patients. Ann Neurol.

[43] Banks WA, Kastin AJ, Komaki G, Arimura A (1993). Passage of pituitary adenylate cyclase activating polypeptide1-27 and pituitary adenylate cyclase activating polypeptide1-38 across the blood- brain barrier. J Pharmacol Exp Ther.

[44] Ghanizada H, Al-Karagholi MA, Arngrim N, Olesen J, Ashina M (2020). PACAP27 induces migraine-like attacks in migraine patients. Cephalalgia..

[45] Erdling A, Sheykhzade M, Maddahi A, Bari F, Edvinsson L (2013). VIP/PACAP receptors in cerebral arteries of rat: Characterization, localization and relation to intracellular calcium. Neuropeptides.

[46] Ghanizada H, Al-Karagholi MA, Arngrim N (2019). Effect of pituitary adenylate cyclase-activating polypeptide-27 on cerebral hemodynamics in healthy volunteers: a 3T MRI study. Peptides..

[47] Schytz HW, Birk S, Wienecke T, Kruuse C, Olesen J, Ashina M (2009). PACAP38 induces migraine-like attacks in patients with migraine without aura. Brain..

[48] Grände G, Labruijere S, Haanes KA, MaassenVanDenBrink A, Edvinsson L (2014). Comparison of the vasodilator responses of isolated human and rat middle meningeal arteries to migraine related compounds. J Headache Pain..

[49] Amin FM, Hougaard A, Schytz HW, Asghar MS, Lundholm E, Parvaiz AI, de Koning PJH, Andersen MR, Larsson HBW, Fahrenkrug J, Olesen J, Ashina M (2014). Investigation of the pathophysiological mechanisms of migraine attacks induced by pituitary adenylate cyclase-activating polypeptide-38. Brain..

[50] Dufes C, Olivier JC, Gaillard F, Gaillard A, Couet W, Muller JM (2003). Brain delivery of vasoactive intestinal peptide (VIP) following nasal administration to rats. Int J Pharm.

[51] Hansen JM, Sitarz J, Birk S, Rahmann AM, Oturai PS, Fahrenkrug J, Olesen J, Ashina M (2006). Vasoactive intestinal polypeptide evokes only a minimal headache in healthy volunteers. Cephalalgia..

[52] Rahmann A, Wienecke T, Hansen JM, Fahrenkrug J, Olesen J, Ashina M (2008). Vasoactive intestinal peptide causes marked cephalic vasodilation, but does not induce migraine. Cephalalgia..

[53] Pellesi L, Al-Karagholi MA, Chaudhry BA (2020). Two-hour infusion of vasoactive intestinal polypeptide induces delayed headache and extracranial vasodilation in healthy volunteers. Cephalalgia.

[54] Dahl A, Russell D, Nyberg-Hansen R, Rootwelt K (1989). Effect of nitroglycerin on cerebral circulation measured by transcranial Doppler and SPECT. Stroke..

[55] Schulz JM, Al-Khazraji BK, Shoemaker JK (2018). Sodium nitroglycerin induces middle cerebral artery vasodilatation in young, healthy adults. Exp Physiol.

[56] Schoonman GG, Van Der Grond J, Kortmann C, Van Der Geest RJ, Terwindt GM, Ferrari MD (2008). Migraine headache is not associated with cerebral or meningeal vasodilatation - a 3T magnetic resonance angiography study. Brain..

[57] Gómez-Vallejo V, Ugarte A, García-Barroso C, Cuadrado-Tejedor M, Szczupak B, Dopeso-Reyes IG, Lanciego JL, García-Osta A, Llop J, Oyarzabal J, Franco R (2016). Pharmacokinetic investigation of sildenafil using positron emission tomography and determination of its effect on cerebrospinal fluid cGMP levels. J Neurochem.

[58] Kruuse C, Frandsen E, Schifter S, Thomsen LL, Birk S, Olesen J (2003). Plasma levels of cAMP, cGMP and CGRP in sildenafil-induced headache. Cephalalgia.

[59] Grände G, Nilsson E, Edvinsson L (2013). Comparison of responses to vasoactive drugs in human and rat cerebral arteries using myography and pressurized cerebral artery method. Cephalalgia..

[60] Kruuse C, Gupta S, Nilsson E, Kruse L, Edvinsson L (2012). Differential vasoactive effects of sildenafil and tadalafil on cerebral arteries. Eur J Pharmacol.

[61] Guo S, Olesen J, Ashina M (2014). Phosphodiesterase 3 inhibitor cilostazol induces migraine-like attacks via cyclic AMP increase. Brain..

[62] Butt JH, Rostrup E, Hansen AS, Lambertsen KL, Kruuse C (2018). Induction of migraine-like headache, but not aura, by cilostazol in patients with migraine with aura. Brain..

[63] Birk S, Kruuse C, Petersen KA, Jonassen O, Tfelt-Hansen P, Olesen J (2004). The phosphodiesterase 3 inhibitor cilostazol dilates large cerebral arteries in humans without affecting regional cerebral blood flow. J Cereb Blood Flow Metab.

[64] Khan S, Amin FM, Christensen CE, Ghanizada H, Younis S, Olinger ACR, de Koning PJH, Larsson HBW, Ashina M (2019). Meningeal contribution to migraine pain: a magnetic resonance angiography study. Brain..

[65] Al-Karagholi MA, Hansen JM, Guo S, Olesen J, Ashina M (2019). Opening of ATP-sensitive potassium channels causes migraine attacks: a new target for the treatment of migraine. Brain..

[66] Al-Karagholi MAM, Ghanizada H, Nielsen CAW (2020). Cerebrovascular effects of glibenclamide investigated using high-resolution magnetic resonance imaging in healthy volunteers. J Cereb Blood Flow Metab.

[67] Al-Karagholi MA, Ghanizada H, Waldorff Nielsen CA (2021). Opening of BKCa channels causes migraine attacks: a new downstream target for the treatment of migraine. Pain.

[68] Al-karagholi MA, Ghanizada H, Amalie C (2020). Opening of BK Ca channels alters cerebral hemodynamic and causes headache in healthy volunteers. Cephalalgia.

[69] Kastin AJ, Akerstrom V, Hackler L, Pan W (2001). Adrenomedullin and the blood-brain barrier. Horm Metab Res.

[70] Banks WA, Kastin AJ (1998). Differential permeability of the blood-brain barrier to two pancreatic peptides: insulin and amylin. Peptides..

[71] Amin FM, Schytz HW (2018). Transport of the pituitary adenylate cyclase-activating polypeptide across the blood-brain barrier: implications for migraine. J Headache Pain..

[72] Birk S, Sitarz JT, Petersen KA, Oturai PS, Kruuse C, Fahrenkrug J, Olesen J (2007). The effect of intravenous PACAP38 on cerebral hemodynamics in healthy volunteers. Regul Pept.

[73] Amin FM, Asghar MS, Guo S, Hougaard A, Hansen AE, Schytz HW, van der Geest RJ, de Koning PJH, Larsson HBW, Olesen J, Ashina M (2012). Headache and prolonged dilatation of the middle meningeal artery by PACAP38 in healthy volunteers. Cephalalgia..

[74] Asghar MS, Hansen AE, Larsson HBW, Olesen J, Ashina M (2012). Effect of CGRP and sumatriptan on the BOLD response in visual cortex. J Headache Pain.

[75] de Vries T, Villalón CM, MaassenVanDenBrink A (2020). Pharmacological treatment of migraine: CGRP and 5-HT beyond the triptans. Pharmacol Ther.

[76] Hostetler ED, Joshi AD, Sanabria-Bohórquez S, Fan H, Zeng Z, Purcell M, Gantert L, Riffel K, Williams M, O’Malley S, Miller P, Selnick HG, Gallicchio SN, Bell IM, Salvatore CA, Kane SA, Li CC, Hargreaves RJ, de Groot T, Bormans G, van Hecken A, Derdelinckx I, de Hoon J, Reynders T, Declercq R, de Lepeleire I, Kennedy WP, Blanchard R, Marcantonio EE, Sur C, Cook JJ, van Laere K, Evelhoch JL (2013). In vivo quantification of calcitonin gene-related peptide receptor occupancy by telcagepant in rhesus monkey and human brain using the positron emission tomography tracer [11C]MK-4232. J Pharmacol Exp Ther.

[77] Christensen SL, Ernstsen C, Olesen J, Kristensen DM (2020). No central action of CGRP antagonising drugs in the GTN mouse model of migraine. Cephalalgia..

[78] Noseda R, Schain AJ, Melo-Carrillo A, Tien J, Stratton J, Mai F, Strassman AM, Burstein R (2020). Fluorescently-labeled fremanezumab is distributed to sensory and autonomic ganglia and the dura but not to the brain of rats with uncompromised blood brain barrier. Cephalalgia..

[79] Eigenbrodt AK, Ashina H, Khan S, Diener HC, Mitsikostas DD, Sinclair AJ, Pozo-Rosich P, Martelletti P, Ducros A, Lantéri-Minet M, Braschinsky M, del Rio MS, Daniel O, Özge A, Mammadbayli A, Arons M, Skorobogatykh K, Romanenko V, Terwindt GM, Paemeleire K, Sacco S, Reuter U, Lampl C, Schytz HW, Katsarava Z, Steiner TJ, Ashina M (2021). Diagnosis and management of migraine in ten steps. Nat Rev Neurol.

[80] Pearlman EM, Wilbraham D, Dennehy EB (2020). Effects of lasmiditan on simulated driving performance: Results of two randomized, blinded, crossover studies with placebo and active controls. Hum Psychopharmacol.

[81] Clemow DB, Johnson KW, Hochstetler HM (2020). Lasmiditan mechanism of action - review of a selective 5-HT 1F agonist. J Headache Pain.

[82] Montagne A, Barnes SR, Sweeney MD, Halliday MR, Sagare AP, Zhao Z, Toga AW, Jacobs RE, Liu CY, Amezcua L, Harrington MG, Chui HC, Law M, Zlokovic BV (2015). Blood-brain barrier breakdown in the aging human Hippocampus. Neuron..

[83] Nation DA, Sweeney MD, Montagne A, Sagare AP, D’Orazio LM, Pachicano M, Sepehrband F, Nelson AR, Buennagel DP, Harrington MG, Benzinger TLS, Fagan AM, Ringman JM, Schneider LS, Morris JC, Chui HC, Law M, Toga AW, Zlokovic BV (2019). Blood-brain barrier breakdown is an early biomarker of human cognitive dysfunction. Nat Med.

[84] Afridi SK, Kaube H, Goadsby PJ (2004). Glyceryl trinitrate triggers premonitory symptoms in migraineurs. Pain..

[85] Maniyar FH, Sprenger T, Monteith T, Schankin C, Goadsby PJ (2014). Brain activations in the premonitory phase of nitroglycerin-triggered migraine attacks. Brain..

[86] Guo S, Vollesen ALH, Olesen J, Ashina M (2016). Premonitory and nonheadache symptoms induced by CGRP and PACAP38 in patients with migraine. Pain..

[87] Al-Karagholi MA-M, Ghanizada H, Nielsen CAW, Hougaard A, Ashina M (2021). Opening of ATP sensitive potassium channels causes migraine attacks with aura. Brain..

[88] Oliver KR, Wainwright A, Edvinsson L, Pickard JD, Hill RG (2002). Immunohistochemical localization of calcitonin receptor-like receptor and receptor activity-modifying proteins in the human cerebral vasculature. J Cereb Blood Flow Metab.

[89] Lennerz JK, Rühle V, Ceppa EP, Neuhuber WL, Bunnett NW, Grady EF, Messlinger K (2008). Calcitonin receptor-like receptor (CLR), receptor activity-modifying protein 1 (RAMP1), and calcitonin gene-related peptide (CGRP) immunoreactivity in the rat trigeminovascular system: differences between peripheral and central CGRP receptor distribution. J Comp Neurol.

[90] Edvinsson JCA, Warfvinge K, Krause DN, Blixt FW, Sheykhzade M, Edvinsson L, Haanes KA (2019). C-fibers may modulate adjacent Aδ-fibers through axon-axon CGRP signaling at nodes of Ranvier in the trigeminal system. J Headache Pain..

[91] Walker CS, Eftekhari S, Bower RL, Wilderman A, Insel PA, Edvinsson L, Waldvogel HJ, Jamaluddin MA, Russo AF, Hay DL (2015). A second trigeminal CGRP receptor: function and expression of the AMY<inf>1</inf> receptor. Ann Clin Transl Neurol.

[92] Tschopp FA, Henke H, Petermann JB, Tobler PH, Janzer R, Hokfelt T, Lundberg JM, Cuello C, Fischer JA (1985). Calcitonin gene-related peptide and its binding sites in the human central nervous system and pituitary. Proc Natl Acad Sci U S A.

[93] Stachniak TJE, Krukoff TL (2003). Receptor activity modifying protein 2 distribution in the rat central nervous system and regulation by changes in blood pressure. J Neuroendocrinol.

[94] Hensley K, Pretorius J, Chan B (2018). PAC1 receptor mRNA and protein distribution in rat and human trigeminal and sphenopalatine ganglia, spinal trigeminal nucleus and in dura mater. Cephalalgia.

[95] Jansen-Olesen I, Baun M, Amrutkar DV, Ramachandran R, Christophersen DV, Olesen J (2014). PACAP-38 but not VIP induces release of CGRP from trigeminal nucleus caudalis via a receptor distinct from the PAC1 receptor. Neuropeptides..

[96] Jolivel V, Basille M, Aubert N, de Jouffrey S, Ancian P, le Bigot JF, Noack P, Massonneau M, Fournier A, Vaudry H, Gonzalez BJ, Vaudry D (2009). Distribution and functional characterization of pituitary adenylate cyclase-activating polypeptide receptors in the brain of non-human primates. Neuroscience..

[97] Joo KM, Chung YH, Kim MK, Nam RH, Lee BL, Lee KH, Cha CI (2004). Distribution of vasoactive intestinal peptide and pituitary adenylate cyclase-activating polypeptide receptors (VPAC1, VPAC2, and PAC1 receptor) in the rat brain. J Comp Neurol.

[98] Ploug KB, Sørensen MA, Strøbech L, Klaerke DA, Hay-Schmidt A, Sheykhzade M, Olesen J, Jansen-Olesen I (2008). KATP channels in pig and human intracranial arteries. Eur J Pharmacol.

[99] Ploug KB, Boni LJ, Baun M, Hay-Schmidt A, Olesen J, Jansen-Olesen I (2008). K ATP channel expression and pharmacological in vivo and in vitro studies of the K ATP channel blocker PNU-37883A in rat middle meningeal arteries. Br J Pharmacol.

[100] Nui K, Saloman JL, Zhang Y, Ro JY (2011). Sex diffferences in the contribution of ATP-sensitive K+ channels in trigeminal ganglia under an acute muscle pain condition. Neuroscience.

[101] Ploug KB, Amrutkar DV, Baun M, Ramachandran R, Iversen A, Lund TM, Gupta S, Hay-Schmidt A, Olesen J, Jansen-Olesen I (2012). K ATP channel openers in the trigeminovascular system. Cephalalgia..

[102] Dunn-Meynell AA, Rawson NE, Levin BE (1998). Distribution and phenotype of neurons containing the ATP-sensitive K+ channel in rat brain. Brain Res.

[103] Sausbier U, Sausbier M, Sailer CA, Arntz C, Knaus HG, Neuhuber W, Ruth P (2006). Ca2+−activated K+ channels of the BK-type in the mouse brain. Histochem Cell Biol.

[104] Wulf-Johansson H, Amrutkar DV, Hay-Schmidt A, Poulsen AN, Klaerke DA, Olesen J, Jansen-Olesen I (2010). Localization of large conductance calcium-activated potassium channels and their effect on calcitonin gene-related peptide release in the rat trigemino-neuronal pathway. Neuroscience..

[105] Poulsen AN, Wulf H, Hay-Schmidt A, Jansen-Olesen I, Olesen J, Klaerke DA (2009). Differential expression of BK channel isoforms and β-subunits in rat neuro-vascular tissues. Biochim Biophys Acta Biomembr.

